# Artificial intelligence‐based echocardiographic assessment for monitoring disease progression in transthyretin cardiac amyloidosis

**DOI:** 10.1002/ejhf.70073

**Published:** 2025-10-16

**Authors:** Lucia Venneri, Alberto Aimo, Aldostefano Porcari, Irem Sezer, Adam Ioannou, Awais Sheikh, Josephine Mansell, Yousuf Razvi, Surabhi Bhaskar Iyer, Ana Martinez‐Naharro, Francesco Bandera, Sze Chi Lim, Matthew Frost, Justin Ezekowitz, Carolyn S.P. Lam, William Moody, Carol Whelan, Helen Lachmann, Ashutosh Wechelakar, Michele Emdin, Philip N. Hawkins, Scott David Solomon, Julian D. Gillmore, Marianna Fontana

**Affiliations:** ^1^ National Amyloidosis Centre, Royal Free Hospital, London University College London London UK; ^2^ Institute of Life Sciences, Scuola Superiore Sant'Anna Pisa Italy; ^3^ Cardiology Division, Fondazione Toscana Gabriele Monasterio Pisa Italy; ^4^ Cardiology Unit, IRCCS MultiMedica Milan Italy; ^5^ Department for Biomedical Sciences for Health University of Milan Milan Italy; ^6^ Us2.ai Singapore Singapore; ^7^ University of Alberta Edmonton Canada; ^8^ National Heart Centre Singapore Singapore; ^9^ Department of Cardiology, Queen Elizabeth Hospital Birmingham Birmingham UK; ^10^ Institute of Cardiovascular Sciences, University of Birmingham Birmingham UK; ^11^ Cardiovascular Division, Brigham and Women's Hospital Harvard Medical School Boston MA USA

**Keywords:** Transthyretin amyloid cardiomyopathy, Cardiac amyloidosis, ATTR, Stroke volume, Echocardiography, Artificial intelligence, Disease progression, Prognosis, Risk stratification

## Abstract

**Aims:**

In transthyretin amyloid cardiomyopathy (ATTR‐CM), reduced stroke volume (SV) portends a poor prognosis. Artificial intelligence (AI) enables rapid, standardized assessment of left ventricular outflow tract velocity‐time integral (LVOT‐VTI), which is a reliable surrogate for SV. We investigated longitudinal changes in AI‐derived LVOT‐VTI as outcome predictors in ATTR‐CM.

**Methods and results:**

Consecutive patients with ATTR‐CM underwent baseline and 12 ± 1 month transthoracic echocardiography between 2007 and 2021. Scans were processed by an AI platform for fully automated measurements including LVOT‐VTI. Changes in echocardiographic variables were related to all‐cause mortality in a landmark analysis using multivariable Cox models adjusting for clinical covariates (age, sex, *TTR* genotype, atrial fibrillation status, New York Heart Association class and National Amyloidosis Centre stage). Time‐dependent receiver‐operating characteristic analysis identified the optimal threshold of LVOT‐VTI change. A total of 752 patients (74 ± 9 years; 88% men; 66% wild‐type) were followed for a median of 3.3 years (interquartile range 2.1–5.0 years), during which 334 (44.4%) died. Among changes in echocardiographic parameters over 12 months, only LVOT‐VTI change remained independently prognostic (adjusted hazard ratio [HR] per 1% decrease 0.994, *p* = 0.025). A ≥5% decrease (*n* = 377 patients, 50%) independently predicted all‐cause mortality (adjusted HR 1.41, 95% confidence interval 1.13–1.76; *p* = 0.003), and improved risk reclassification (integrated discrimination improvement = 0.012; continuous net reclassification improvement = 0.21, both *p* < 0.001).

**Conclusions:**

A ≥5% decrease of AI‐derived LVOT‐VTI over 12 months, a simple indicator of SV loss, is independently associated with worse outcome in ATTR‐CM. Routine monitoring of this automated AI metric may guide earlier therapeutic escalation and is a possible endpoint for future trials.

## Introduction

Transthyretin amyloid cardiomyopathy (ATTR‐CM) is a progressive, life‐threatening condition characterized by extracellular amyloid deposition in the interstitial spaces of the heart.^1^ ATTR‐CM can be classified into the hereditary form which is associated with mutations in the *TTR* gene (ATTRv), and the wild‐type form (ATTRwt) which is late‐onset, with a male predominance. In recent years, advances in diagnostics techniques, increased awareness and the availability of effective treatments have led to a significant rise in the diagnoses of ATTR‐CM.^2^

Beyond tafamidis, another transthyretin (TTR) stabilizer (acoramidis) and the TTR silencer vutrisiran are now licensed for ATTR‐CM, and multiple Phase 3 trials are underway.^3^ As options broaden, rigorous assessment of treatment response is imperative.^3^ Patients who fail to improve, or whose cardiac function declines, could be considered for other treatments, making the early identification of prognostic indicators essential.

Echocardiography is the first‐line imaging modality in patients with ATTR‐CM.^4^ While its diagnostic and prognostic utility is well established, its role in serial assessments for tracking disease progression at an individual level remains limited due to the inherent variability in ultrasonographic measurements among and within operators. Most notably, reduced stroke volume (SV) portends a poor prognosis in ATTR‐CM.^5^ Left ventricular outflow tract velocity‐time integral (LVOT‐VTI) is a reliable surrogate for SV, yet its routine use has been limited by the time‐consuming, operator‐dependent nature of manual measurements. A Food and Drug Administration‐cleared platform now delivers fully automated, artificial intelligence (AI)‐derived LVOT‐VTI, enabling rapid and standardized assessment of SV.^6^ Whether longitudinal changes in this AI‐derived metric carry independent prognostic significance in ATTR‐CM, however, has not been established.

In this study, we examined the prognostic value of changes in a broad range of AI‐derived echocardiographic parameters, aiming to establish a criterion for disease progression in ATTR‐CM.

## Methods

### Patient population and follow‐up

We evaluated consecutive patients with ATTR‐CM undergoing a baseline echocardiogram as part of their diagnostic work‐up and then 12 ± 1 months later, in the period between January 2007 and August 2021. ATTR‐CM was diagnosed through tissue biopsy until 2016, and according to the algorithm for non‐biopsy diagnosis^7^ from 2016 onwards. All patients provided written informed consent for their data to be analysed and published, in accordance with the Declaration of Helsinki and approval from the Royal Free Hospital ethics committee (ref: 21/PR/0620). Follow‐up began with the 12‐month echocardiogram and was censored on 30 September 2024. Mortality data were obtained via the UK Office of National Statistics.

### Echocardiography

Two‐dimensional transthoracic echocardiographic examinations were performed by experienced operators using GE Vivid E9 ultrasound machine equipped with a 5S probe. The echocardiograms were uploaded to the Us2.ai platform, which automates the entire workflow of echocardiographic analysis and interpretation.^8^

Automated LVOT‐VTI is derived from the Doppler envelope without using device cursor metadata. LVOT‐VTI was generated only when the Doppler image passed the workflow's predefined quality criteria and highest‐confidence selection. Automated analysis is operator‐independent and has shown lower variability than human–human comparisons in prior validation studies.^8^, ^9^

### Statistical analysis

Data were analysed using SPSS (version 27.0. IBM Corp., Armonk, NY, USA), R (version 4.2.2) and Python 3.10. All continuous variables were tested for normality using the Shapiro–Wilk test. Data are presented as mean ± standard deviation if normally distributed and as median and interquartile range if non‐normally distributed. Continuous variables were compared through the independent sample *t*‐test or the Mann–Whitney U test for normally and non‐normally distributed variables, respectively. Categorical data are presented as absolute numbers and percentages and compared using the Chi‐square test. The prognostic value of percentage changes in echocardiographic variables was assessed using univariable and multivariable Cox regression; missing data were excluded (i.e. not imputed). In multivariable analysis, univariable predictors were evaluated on top of a model including age, sex, *TTR* genotype (wild‐type, V122I, or other variant), atrial fibrillation status (atrial fibrillation vs. sinus rhythm or paced rhythm), New York Heart Association (NYHA) class and National Amyloidosis Centre (NAC) stage.^10^ The proportional hazards assumption was checked. Multicollinearity was excluded through the variance inflation factor, with a threshold of 5. To characterize the shape of the association between the percent change in LVOT‐VTI and subsequent clinical events, we fitted the exposure as a restricted cubic spline within a univariable Cox proportional‐hazards model, with four knots placed at the 5th, 35th, 65th and 95th percentiles of the LVOT‐VTI distribution. The discriminatory ability of percent change in LVOT‐VTI (%ΔLVOT‐VTI) was assessed with a receiver operating characteristic (ROC) curve. The ROC curve was computed twice, once using the raw values and once using their sign‐inverted counterparts (−%ΔLVOT‐VTI). For each direction the Youden index was calculated at every unique predictor value, and the cut‐off that maximized J (sensitivity + specificity − 1) was identified. The direction yielding the larger maximal J was retained as the provisional ‘optimal’ threshold. To quantify the stability of that threshold and its operating characteristics we performed 1000 bootstrap resamples.

Landmark survival analysis was carried out to assess the relationship between the cut‐off value identified (LVOT‐VTI decrease ≥5%) at the 1‐year follow‐up timepoint, and all‐cause mortality from the 1‐year timepoint onwards. This was checked through Kaplan–Meier survival curves with log‐rank analysis, multivariable Cox regression analysis, and reclassification analysis. The prognostic value across subgroups was visually represented through a Forest plot; likelihood ratio χ^2^ tests provided two‐sided *p*‐values for interaction. Predictors of the LVOT‐VTI decrease ≥5% were searched through uni‐ and multivariable logistic regression analysis. Statistical significance was defined as *p* < 0.05.

## Results

### Patient population and follow‐up

The flowchart of patient selection is reported in online supplementary *Figure* *S1*. The final study population included 752 patients, with 495 (65.8%) having ATTRwt‐CM and 257 (34.2%) having ATTRv‐CM (145 with V122I; 67 with T60A, 45 with other mutations). Patients were aged 74 ± 9 years, and 661 (87.9%) were male. Most patients had NYHA class I or II symptoms (82.4% overall), and either NAC stage 1 (*n* = 424, 57%) or stage 2 (*n* = 242, 32%) disease. Furthermore, 49.6% were on atrial fibrillation at the time of the first echo (*Table* *1*). At baseline, 32 patients (4%) were on tafamidis, 51 (7%) on gene silencers, and 79 (11%) were enrolled in clinical trials.

**Table 1:** Main baseline clinical features of the study population

Variables	Total (*n* = 752)	Missing data, *n* (%)
Age (years)	74 ± 9	0 (0)
Male sex, *n* (%)	661 (87.9)	0 (0)
Genotype, *n* (%)		
wt	495 (65.8)	0 (0)
V122I	145 (19.3)	0 (0)
Non‐V122I	112 (14.9)	0 (0)
Comorbidities, *n* (%)		
Atrial fibrillation^a^	373 (49.6)	0 (0)
Ischaemic heart disease	139 (18.5)	0 (0)
Diabetes mellitus	119 (15.8)	0 (0)
Hypertension	222 (29.5)	0 (0)
NYHA class^b^, *n* (%)		
I	127 (17.6)	30 (4)
II	468 (64.8)	
III	118 (16.3)	
IV	9 (1.2)	
NAC stage^b^, *n* (%)		
1	424 (56.8)	6 (1)
2	242 (32.4)	
3	80 (10.7)	
NT‐proBNP (ng/L)	2360 (1251–4267)	7 (1)
eGFR (ml/min/1.73 m^2^)	62 (50–76)	2 (0)
Therapies, *n* (%)		
Loop diuretic	583 (77.5)	0 (0)
MRA	341 (45.3)	0 (0)
ACEi/ARB/ARNI	463 (62.6)	0 (0)
SGLT2i	6 (0.8)	0 (0)
Beta‐blocker	422 (56.1)	0 (0)
PPM	91 (12.1)	0 (0)
ICD	22 (2.9)	0 (0)
CRT	26 (3.5)	0 (0)

ACEi, angiotensin‐converting enzyme inhibitor; ARB, angiotensin receptor blocker; ARNI, angiotensin receptor–neprilysin inhibitor; CRT, cardiac resynchronization therapy; eGFR, estimated glomerular filtration rate; ICD, implantable cardioverter‐defibrillator; MRA, mineralocorticoid receptor antagonist; NAC, National Amyloidosis Centre; NT‐proBNP, N‐terminal pro‐B‐type natriuretic peptide; NYHA, New York Heart Association; PPM, permanent pacemaker; SGLT2i, sodium–glucose co‐transporter 2 inhibitor; wt, wild‐type.

^a^ Defined as presence of atrial fibrillation on baseline echocardiography.

^b^ For NYHA class and NAC stage, percentages were calculated out of the patients with available values.

### Change in echocardiographic parameters over 1 year

At baseline, patients had a median interventricular septal thickness of 14 mm (interquartile range 12–16), and left ventricular mass of 229 g (185–275). Left ventricular systolic function was mildly depressed, with median LVEF 49% (41–58), and median global longitudinal strain of −14.5% (−17.2 to −11.3). LVOT‐VTI was 16 cm (13–20). Right ventricular function was also mildly depressed, with a fractional area change of 29% (22–36), and a tricuspid annular plane systolic excursion (TAPSE) of 16 mm (12–20). When considering percent changes from baseline to 12 months, the most prominent changes were in fractional area change (−5.8%), E/e' ratio (+4.8%), and LVOT‐VTI (−4.5%) (*Table* *2*).

**Table 2:** Main echocardiographic findings at baseline and 12 months

Variables	Baseline echo	12‐month echo	Δ%
IVS (mm)	14 (12–16)	14 (13–16)	+2.2 (−7.2 to 11.1)
PW (mm)	14 (12–17)	14 (12–17)	+2.3 (−11.5 to 17.3)
RWT	0.67 (0.55–0.81)	0.67 (0.56–0.83)	+1.9 (−14.1 to 19.9)
LV mass (g)	229 (185–275)	236 (190–282)	+2.7 (−10.3 to 17.5)
LVEF (%)	49 (41–58)	48 (38–57)	−1.3 (−17.0 to 12.4)
LV GLS (%)	−14.5 (−17.2 to −11.3)	−14.4 (−17.1 to −11.1)	−1.6 (−18.1 to 21.5)
LVOT‐VTI (cm)	16 (13–20)	16 (12–19)	−4.5 (−18.8 to 11.4)
E/e' ratio	16 (12–20)	16 (13–21)	+4.8 (−8.8 to 23.4)
TAPSE (mm)	16 (12–20)	16 (13–20)	−2.5 (−24.0 to 29.1)
RV FAC (%)	29 (22–36)	28 (19–35)	−5.8 (−37.8 to 30.2)
sPAP (mmHg)	38 (31–46)	39 (33–47)	+3.4 (−15.0 to 28.0)

FAC, fractional area change; GLS, global longitudinal strain; IVS, interventricular septum; LV, left ventricular; LVEF, left ventricular ejection fraction; LVOT‐VTI, left ventricular outflow tract velocity‐time integral; PW, posterior wall; RV, right ventricular; RWT, relative wall thickness; sPAP, systolic pulmonary artery pressure; TAPSE, tricuspid annular plane systolic excursion.

### Changes in echocardiographic parameters and outcome

The median follow‐up after the 12‐month echocardiogram was 3.3‐years (interquartile range 2.1–5.0 years). During this interval, 334 patients (44.4%) died. Most baseline echocardiographic parameters predicted all‐cause mortality, but none of them had independent prognostic significance in a model including univariable predictors and age, sex, *TTR* genotype, atrial fibrillation status, NYHA class and NAC stage (online supplementary *Table* *S1*).

We then focused on changes from baseline to 12‐month echocardiogram. In univariable Cox regression analysis, the only echocardiographic parameters whose changes were prognostically significant were LVOT‐VTI and TAPSE (*Table* *3*). After adjustment for age, sex, *TTR* genotype, atrial fibrillation status, NYHA class and NAC stage, only changes in LVOT‐VTI retained independent prognostic value (*Table* *3*). The independent prognostic value of changes in LVOT‐VTI was confirmed when therapies (tafamidis vs. no tafamidis therapy; tafamidis or gene silencers vs. none of these therapies) were forced into the multivariable model (data not shown). Additionally, changes in LVOT‐VTI did not differ significantly in patients on tafamidis versus the other patients (*p* = 0.830), and even in those on tafamidis or gene silencers versus the other patients (*p* = 0.403).

**Table 3:** Percent changes (Δ%) in echocardiographic parameters and mortality: Cox regression analysis

Δ%	Univariable			Multivariable^a^		
	HR	95% CI	*p*‐value	HR	95% CI	*p*‐value
IVS	–	–	0.781	–	–	–
PW	–	–	0.073	–	–	–
RWT	–	–	0.663	–	–	–
LV mass	–	–	0.269	–	–	–
LVEF	–	–	0.597	–	–	–
LV GLS	–	–	0.907	–	–	–
LVOT‐VTI	0.994	0.989–0.998	0.006	0.994	0.989–0.999	0.025
E/e' ratio	–	–	0.133	–	–	–
TAPSE	0.995	0.991–0.998	0.004	–	–	0.984
RV FAC	–	–	0.370	–	–	–
sPAP	–	–	0.100	–	–	–

CI, confidence interval; FAC, fractional area change; GLS, global longitudinal strain; HR, hazard ratio; IVS, interventricular septum; LV, left ventricular; LVEF, left ventricular ejection fraction; LVOT‐VTI, left ventricular outflow tract velocity‐time integral; PW, posterior wall; RV, right ventricular; RWT, relative wall thickness; sPAP, systolic pulmonary artery pressure; TAPSE, tricuspid annular plane systolic excursion.

HR and 95% CI values are reported only for significant *p*‐values.

^a^ Model including age, sex, classification according to the *TTR* genotype (wild‐type, V122I, other variant), atrial fibrillation status (atrial fibrillation at the time of first echo vs. sinus rhythm or paced rhythm), New York Heart Association class and National Amyloidosis Centre stage.

**Figure 1:**
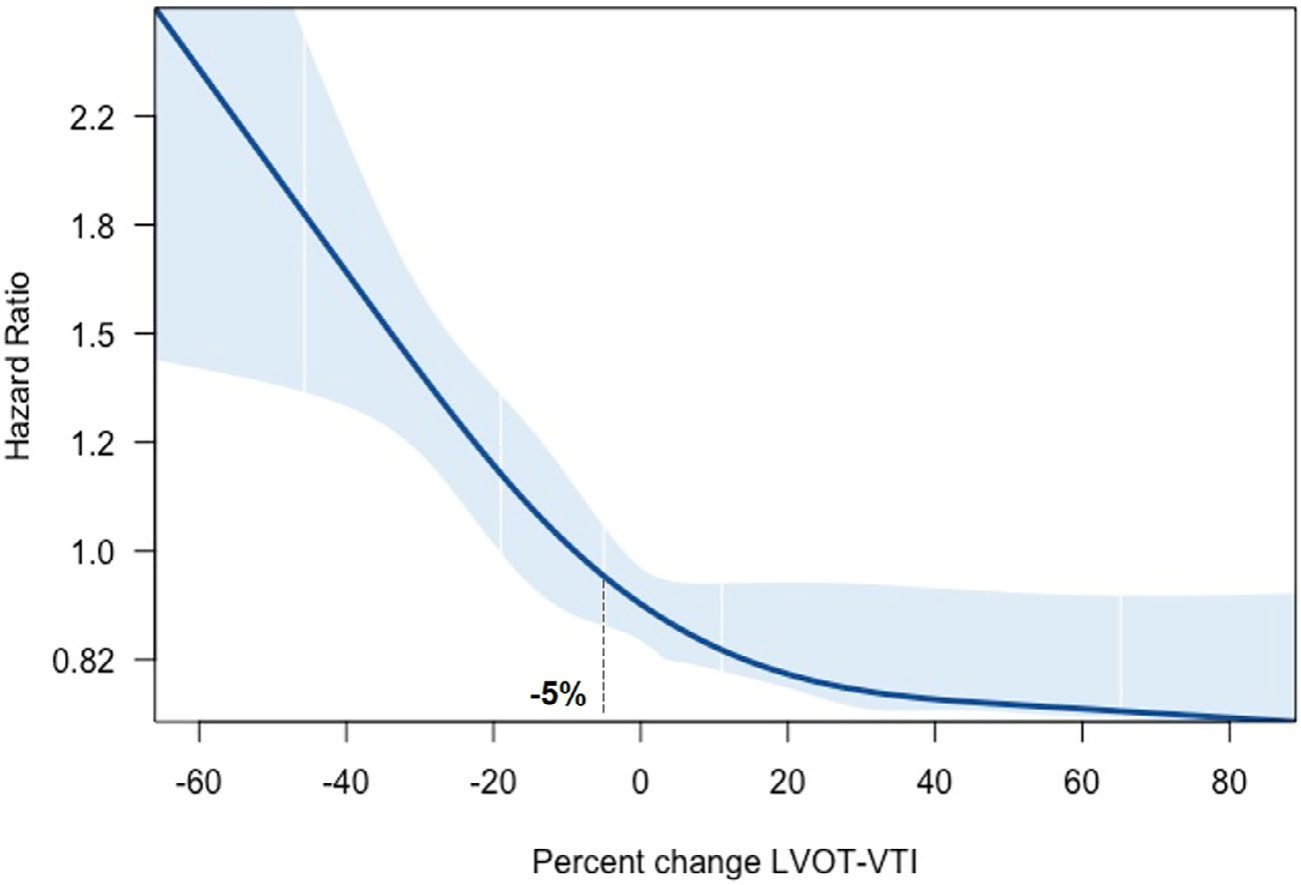
Changes in left ventricular outflow tract velocity‐time integral (LVOT‐VTI) and risk of all‐cause mortality: spline curve analysis. The figure shows the unadjusted continuous association between %ΔLVOT‐VTI and mortality (reference = 0% change). The dashed line at −5% denotes the threshold used for subsequent dichotomized analyses; the adjusted hazard ratio for the comparison ≥5% decrease versus <5% decrease (hazard ratio 1.41) is estimated in the Cox model and is not depicted here.

**Figure 2:**
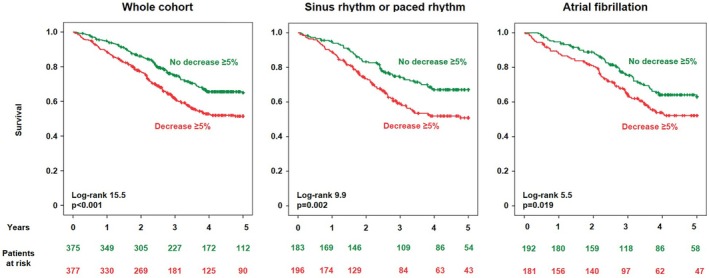
Left ventricular outflow tract velocity‐time integral (LVOT‐VTI) decrease ≥5% over 1 year and outcome. Survival curves for the whole cohort and for the subgroups on sinus rhythm or paced rhythm on atrial fibrillation at the time of baseline echo are provided.

**Figure 3:**
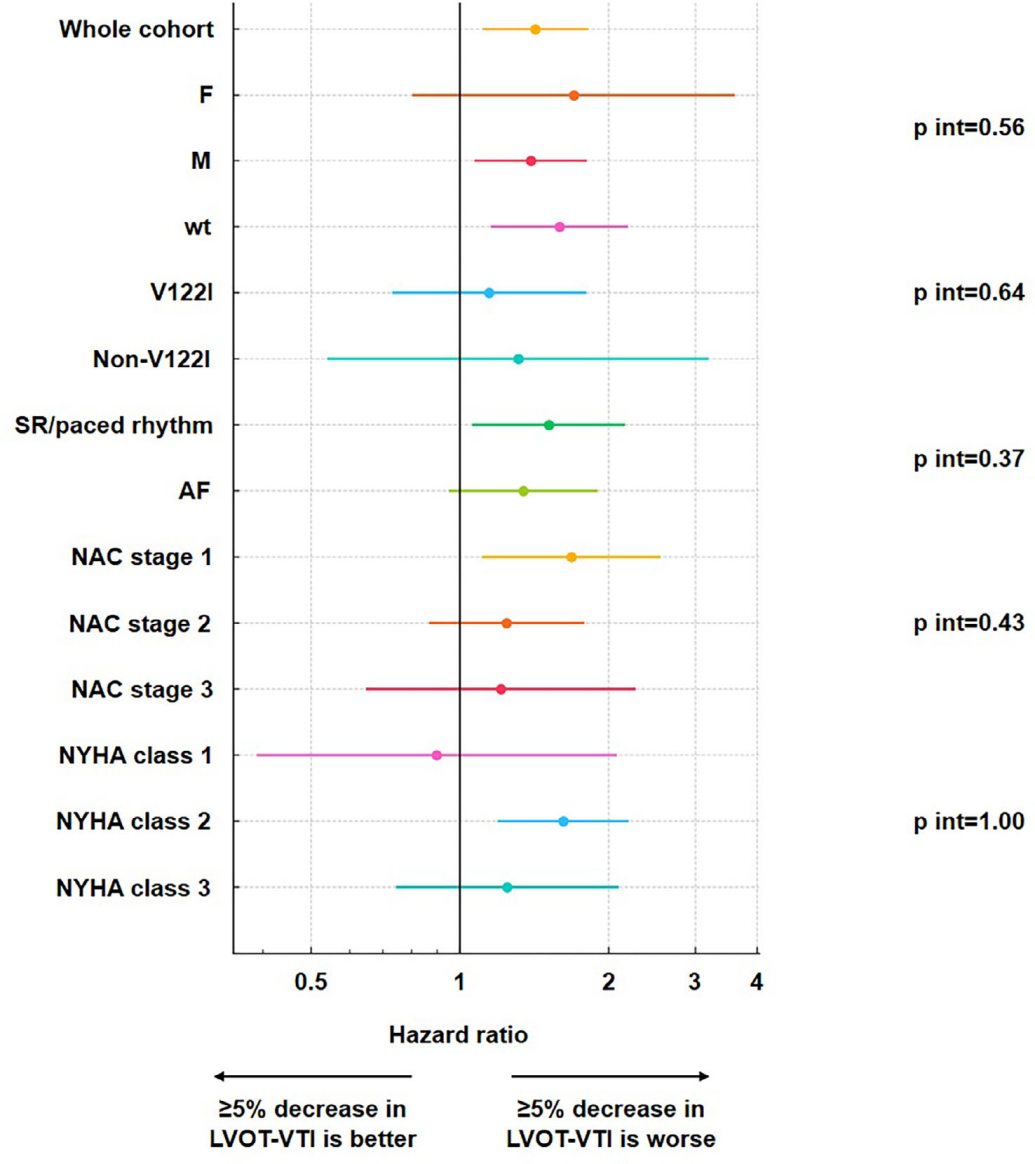
Left ventricular outflow tract velocity‐time integral (LVOT‐VTI) decrease ≥5% over 1 year and outcome: subgroup analysis. Forest plot analysis. The prognostic model included age, sex, *TTR* genotype, atrial fibrillation (AF) status, New York Heart Association (NYHA) class and National Amyloidosis Centre (NAC) stage. p int, *p*‐value for interaction; SR, sinus rhythm; wt, wild‐type.

The risk of mortality increased exponentially as LVOT‐VTI declined, with an inflection point at roughly a 5% decrease (*Figure* *1*). Accordingly, the optimal cut‐off derived from Youden's J was a ≥5% decrease in LVOT‐VTI (online supplementary *Table* *S2*). A reduction in LVOT‐VTI ≥5% predicted all‐cause mortality (log‐rank 15.5, *p* < 0.001) with an early divergence of the survival curves (*Figure* *2*). After adjustment for age, sex, *TTR* genotype, atrial fibrillation status, NYHA class and NAC stage, LVOT‐VTI decrease ≥5% remained predictive of all‐cause mortality (hazard ratio 1.41, 95% confidence interval 1.13–1.76; *p* = 0.003). Moreover, adding this threshold to the adjusted model significantly improved risk reclassification (integrated discrimination improvement = 0.012; continuous net reclassification improvement = 0.21, both *p* < 0.001). The prognostic value of LVOT‐VTI decrease ≥5% was consistent across subgroups (*Figure* *3*).

### Left ventricular outflow tract velocity‐time integral decrease: predictors and correlates

Half of patients (*n* = 377, 50.1%) displayed a LVOT‐VTI decrease ≥5%. At baseline, they had a higher NAC stage, were more often on loop diuretics and mineralocorticoid receptor antagonists (MRA). They had also higher LVOT‐VTI and TAPSE values (*Table* *4*). Among baseline characteristics, age, NAC stage, estimated glomerular filtration rate, loop diuretic and therapy with MRA, and LVOT‐VTI predicted a LVOT‐VTI decrease ≥5%. In a multivariable model including all the univariable predictors (except for estimated glomerular filtration rate to avoid multicollinearity), the only independent predictors of LVOT‐VTI decrease ≥5% were NAC stage, therapy with MRA, and baseline LVOT‐VTI (online supplementary *Table* *S3*). With respect to changes from baseline to 12 months, a LVOT‐VTI decrease ≥5% was associated with a decline in LVEF, left ventricular global longitudinal strain, and TAPSE (online supplementary *Table* *S4*).

**Table 4:** Patients developing a decrease in left ventricular outflow tract velocity‐time integral ≥5% over 12 months: baseline characteristics

Variables	LVOT‐VTI decrease ≥5% (*n* = 377, 50.1%)	No LVOT‐VTI decrease ≥5% (*n* = 375, 49.9%)	*p*‐value
Age (years)	76 (70–80)	75 (69–80)	0.083
Male sex, *n* (%)	337 (89.4)	324 (86.4)	0.209
Genotype, *n* (%)			
wt	250 (66.3)	245 (65.3)	0.060
V122I	26 (6.9)	41 (10.9)	
Non‐V122I	101 (26.8)	89 (23.7)	
Comorbidities			
Atrial fibrillation^a^	181 (48.0)	192 (51.2)	0.382
Ischaemic heart disease	74 (19.6)	65 (17.3)	0.417
Diabetes mellitus	62 (16.4)	57 (15.2)	0.640
Hypertension	123 (32.6)	99 (26.4)	0.061
NYHA class^b^, *n* (%)			
I	56 (15.5)	71 (19.7)	0.389
II	239 (66.2)	229 (63.4)	
III	60 (16.6)	58 (16.1)	
IV	6 (1.7)	3 (0.8)	
NAC stage^b^, *n* (%)			
1	202 (53.9)	222 (59.8)	**0.030**
2	122 (32.5)	120 (32.3)	
3	51 (13.6)	29 (7.8)	
NT‐proBNP (ng/L)	2424 (1281–4165)	2309 (1092–4301)	0.455
eGFR (ml/min/1.73 m^2^)	59 (47–72)	66 (53–80)	**<0.001**
Therapies, *n* (%)			
Loop diuretic	306 (81.2)	277 (73.9)	**0.016**
MRA	188 (49.9)	153 (40.8)	**0.013**
ACEi/ARB/ARNI	240 (63.7)	223 (59.4)	0.321
SGLT2i	1 (0.3)	5 (1.3)	0.100
Beta‐blocker	213 (56.5)	209 (55.7)	0.833
PPM	48 (12.7)	43 (11.5)	0.595
ICD	8 (2.1)	14 (3.7)	0.190
CRT	14 (3.7)	12 (3.2)	0.768
Baseline echo			
IVS (mm)	14 (13–16)	14 (12–16)	0.652
PW (mm)	14 (12–16)	14 (11–17)	0.143
RWT	0.67 (0.56–0.81)	0.66 (0.53–0.80)	0.148
LV mass (g)	234 (190–278)	224 (182–272)	0.201
LVEF (%)	50 (42–59)	48 (40–57)	0.180
LV GLS (%)	−14.7 (−17.7 to −11.8)	−14.1 (−16.9 to −10.8)	0.154
LVOT‐VTI (cm)	18 (15–21)	15 (12–18)	**<0.001**
E/e' ratio	16 (13–20)	16 (12–19)	0.150
TAPSE (mm)	17 (13–21)	16 (12–19)	**0.049**
RV FAC (%)	29 (22–36)	29 (22–36)	0.856
sPAP (mmHg)	38 (31–45)	39 (33–47)	0.130

ACEi, angiotensin‐converting enzyme inhibitor; ARB, angiotensin receptor blocker; ARNI, angiotensin receptor–neprilysin inhibitor; CRT, cardiac resynchronization therapy; eGFR, estimated glomerular filtration rate; FAC, fractional area change; GLS, global longitudinal strain; ICD, implantable cardioverter‐defibrillator; IVS, interventricular septum thickness; LV, left ventricular; LVEF, left ventricular ejection fraction; LVOT‐VTI, left ventricular outflow tract velocity‐time integral; MRA, mineralocorticoid receptor antagonist; NT‐proBNP, N‐terminal pro‐B‐type natriuretic peptide; PPM, permanent pacemaker; PW, posterior wall; RV, right ventricular; RWT, relative wall thickness; sPAP, systolic pulmonary artery pressure; SGLT2i, sodium–glucose co‐transporter 2 inhibitor; SV, stroke volume; TAPSE, tricuspid annular plane systolic excursion; wt, wild‐type.

^a^ Defined as presence of atrial fibrillation at the time of first echocardiographic exam.

^b^ For NYHA class and NAC stage, percentages were calculated out of the patients with available values.

## Discussion

In the present study, we analysed baseline and 12‐month transthoracic echocardiograms in 752 consecutive patients with ATTR‐CM using a clinically available AI platform. The study demonstrated that: (i) although almost every structural and Doppler metric at diagnosis related to outcome, serial analysis revealed that the only change that retained independent prognostic value after adjustment for age, sex, genotype, rhythm, NYHA class and NAC stage was a decline in the AI‐derived LVOT‐VTI; (ii) a fall of at least 5% over 1 year was common, occurring in half of the patients and conferred a 41% excess risk of all‐cause mortality over the subsequent 5 years; and (iii) incorporating this single binary variable into the multivariable model significantly improved two metrics of risk reclassification, underscoring its incremental information content beyond established clinical staging (*Graphical Abstract*).

Transthyretin amyloid cardiomyopathy is characterized by relentless extracellular fibril deposition that stiffens the ventricle, shortens diastolic filling, lowers end‐diastolic volume and progressively erodes SV even when ejection fraction appears preserved.^11^ LVOT‐VTI captures the integral of systolic flow through a conduit whose cross‐sectional area is essentially constant for a given patient; it therefore mirrors beat‐to‐beat SV without the geometric assumptions and squared‐radius amplification of conventional Doppler or Simpson biplane calculations.^12^ The AI pipeline averages multiple beats, applies uniform border detection and automatically excludes poor‐quality cycles, achieving a reproducibility unlikely to be matched by manual tracing (Ioannou A., unpublished data). In our dataset the median AI‐derived LVOT‐VTI at baseline was 16 cm (interquartile range 13–20) and on average declined by 4.5% at 12 months. The direction and magnitude of change seemed to be particularly predictive. This observation is in line with previous heart failure studies in which VTI trajectories outperformed conventional volumetric or fractional indices,^13^, ^14^ but we extend that concept to the restrictive haemodynamics of ATTR‐CM, for which a reproducible cut‐off has been lacking so far.

The prognostic information conveyed by AI‐derived LVOT‐VTI decline proved robust across atrial fibrillation versus other rhythms (i.e. sinus rhythm or paced rhythm), and was paralleled by a steeper deterioration in ejection fraction, global longitudinal strain and TAPSE among patients crossing the 5% threshold. Baseline correlates of subsequent decline included more advanced NAC stage, therapy with MRAs and, paradoxically, higher starting LVOT‐VTI, suggesting that patients with initially greater SV values have greater room for worsening as amyloid infiltration progresses.

Our findings refine earlier work linking static forward‐flow surrogates such as SV index or cardiac index to prognosis in amyloidosis cohorts^13^ and align with previous reports on the prognostic value of SV changes assessed manually by expert readers.^5^ However, in current clinical practice, echocardiography—despite being widely available and routinely repeated every 12 months in patients with ATTR‐CM—is not effectively used to monitor disease progression over time, largely due to operator‐dependent variability in measurements. By enabling fully automated, standardized quantification of LVOT‐VTI, AI overcomes these limitations and offers a simple, geometry‐free metric that reliably reflects SV. A decline of ≥5% in this parameter provides clinicians with an objective and reproducible signal of worsening haemodynamics, which could prompt earlier escalation of therapy or more intensive heart failure management. Moreover, given its mechanistic relevance, repeatability, and now‐demonstrated prognostic significance, a ≥5% reduction in AI‐derived LVOT‐VTI could serve as an exploratory endpoint in clinical trials evaluating treatment response in ATTR‐CM.^14^

Several limitations must be acknowledged. Generalizability is constrained by the retrospective, single‐centre design and the enrolment window (2007–2021), during which adoption of disease‐modifying ATTR‐CM therapies was evolving. The ≥5% AI‐LVOT‐VTI threshold was internally derived and requires external, prospective validation—particularly in contemporary cohorts treated with TTR stabilizers or silencers. Medication data were captured only at baseline and did not systematically include disease‐modifying agents or time‐updated changes; thus we could not perform the requested exploratory validation in a treated subset, model therapy intensification, or infer causal effects. Although baseline MRA use was associated with subsequent LVOT‐VTI decline, confounding by indication and other residual confounding cannot be excluded. Methodologically, the algorithm averages quality‐checked beats but cannot yet systematically discard post‐extrasystolic cycles (potential bias with frequent ectopy), and right atrial area image quality was occasionally suboptimal. We did not repeat manual LVOT‐VTI re‐measurement in this cohort, although prior work supports comparability and lower variability with the same Doppler workflow.^8^, ^9^ Finally, we used all‐cause mortality without adjudicating cardiovascular death and did not perform cause‐specific or competing‐risk analyses, which may dilute cardiovascular effect estimates and limits mechanistic specificity.

In conclusion, fully automated analysis of paired echocardiograms shows that a fall of at least 5% in AI‐derived LVOT‐VTI over 12 months is a simple, reproducible and independent harbinger of 5‐year mortality in ATTR‐CM. This geometry‐free surrogate of SV summarizes the haemodynamic consequences of ongoing fibril deposition more effectively than any other serial echocardiographic metric and can be integrated effortlessly into routine or remote follow‐up. Prospective validation across diverse clinical settings and embedding the metric into treatment algorithms could establish AI‐derived LVOT‐VTI decline as a pragmatic indicator of disease progression and therapeutic response in ATTR‐CM.

### Funding

This work was supported by a fellowship funded by an unrestricted grant from AstraZeneca. The company had no involvement in the design, conduct, analysis, or reporting of the study.

**Conflict of interest**: none declared.

## Supporting information


**Appendix S1.** Supporting Information.
