# Supplementary material for: Artificial intelligence‐based echocardiographic assessment for monitoring disease progression in transthyretin cardiac amyloidosis
Source: Eur J Heart Fail. 2025 Oct 16;27(12):3392–400. doi: 10.1002/ejhf.70073 (PMC12803646; doi:10.1002/ejhf.70073)
Supplement: Supplementary file 1 — Appendix S1. Supporting Information. [file EJHF-27-3392-s001.docx]

**Supplemental material**

**AI-Based Echocardiographic Assessment for Monitoring Disease Progression in Transthyretin Cardiac Amyloidosis**

*Lucia Venneri^1^ MD PhD, *Alberto Aimo^2 3^ MD PhD, Aldostefano Porcari^1^ MD, Irem Sezer^1^ MD, Adam Ioannou^1^ MBBS PhD, Awais Sheikh^1^ MBChB, Josephine Mansell^1^ MBBS, BMedSci, Yousuf Razvi^1^ MBChB, Surabhi Bhaskar Iyer^1^, Ana Martinez-Naharro^1^ MD PhD , Francesco Bandera MD PhD^4 5^, Sze Chi Lim^6^, Matthew Frost^6^, Justin Ezekowitz^7^, Carolyn SP Lam^8^, William Moody^9 10^ MD, Carol Whelan^1^ MD, Helen Lachmann^1^ MD, Ashutosh Wechelakar^1^ MD, Michele Emdin^2 3^ MD PhD, Philip N Hawkins^1^ MD PhD, Scott David Solomon MD^11^, •Julian D Gillmore^1^  MD PhD, •Marianna Fontana^1^  MD PhD

1. National Amyloidosis Centre, Royal Free Hospital, London, University College London, London, United Kingdom
2. Institute of Life Sciences, Scuola Superiore Sant’Anna, Pisa, Italy
3. Cardiology Division, Fondazione Toscana Gabriele Monasterio, Pisa, Italy
4. Cardiology Unit, IRCCS MultiMedica, Milan, Italy
5. Department for Biomedical Sciences for Health, University of Milano, Milan, Italy
6. Us2.ai, Singapore, Singapore
7. University of Alberta, Edmonton, Canada
8. National Heart Centre Singapore & Duke- National University of Singapore, Singapore
9. Department of Cardiology, Queen Elizabeth Hospital Birmingham, Birmingham, United Kingdom
10. Institute of Cardiovascular Sciences, University of Birmingham, Edgbaston, Birmingham, United Kingdom
11. Cardiovascular Division, Brigham and Women's Hospital, Harvard Medical School, Boston, Massachusetts, USA

*Lucia Venneri and Alberto Aimo should be considered co-first author for this work

•Julian Gillmore and Marianna Fontana contributed equally to this work.

**Supplemental Figure 1. Flowchart of patient selection.**


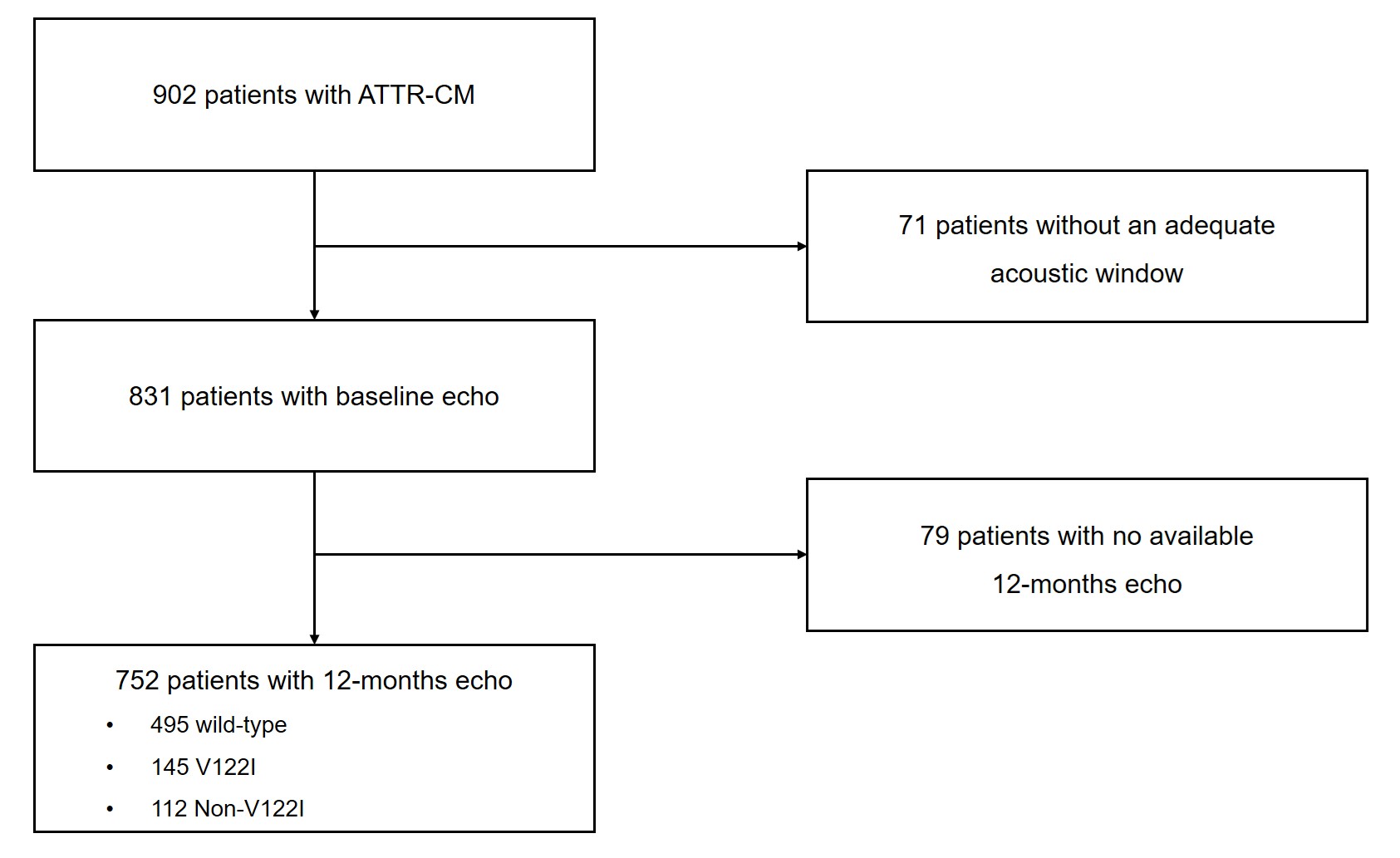


ATTR-CM, amyloid transthyretin cardiomyopathy.

**Supplemental Table 1. Baseline echocardiographic parameters and mortality: Cox regression analysis.**

|  | **Univariable** | | | **Multivariable*** | | |
| --- | --- | --- | --- | --- | --- | --- |
|  | **p** | **HR** | **95% CI** | **p** | **HR** | **95% CI** |
| IVS | <0.001 | 1.08 | 1.04-1.13 | 0.608 | - | - |
| PW | <0.001 | 1.07 | 1.04-1.10 | - | - | - |
| RWT | <0.001 | 3.34 | 1.98-5.63 | 0.747 | - | - |
| LV mass | <0.001 | 1.00 | 1.00-1.01 | 0.080 | - | - |
| LVEF | <0.001 | 0.98 | 0.96-0.99 | - | - | - |
| LV GLS | <0.001 | 1.10 | 1.06-1.15 | 0.800 | - | - |
| LVOT-VTI | <0.001 | 0.95 | 0.93-0.97 | 0.831 | - | - |
| E/e’ ratio | <0.001 | 1.05 | 1.03-1.07 | 0.579 | - | - |
| TAPSE | 0.257 | - | - | - | - | - |
| RV FAC | 0.011 | 0.99 | 0.97-1.00 | 0.981 | - | - |
| sPAP | 0.302 | - | - | - | - | - |

* Model including age, sex, classification according to the *TTR* genotype (wild-type, V122I, other variant), atrial fibrillation status (atrial fibrillation at the time of first echo vs. sinus rhythm or paced rhythm), New York Heart Association class and National Amyloidosis Centre stage. Hazard ratio (HR) and 95% confidence interval (CI) values are reported only for significant p values. Some of the univariable predictors were not entered into the multivariable model to avoid multicollinearity. FAC, fractional area change; GLS, global longitudinal strain; IVS, interventricular septum; LV, left ventricular; LVEF, left ventricular ejection fraction; LVOT-VTI, left ventricular outflow tract-velocity time interval; PW, posterior wall; RV, right ventricular; RWT, relative wall thickness; sPAP, systolic pulmonary artery pressure; SV, stroke volume; TAPSE, tricuspid annular plane systolic excursion.

**Supplemental Table 2. Identification of a cut-off for percent change in left ventricular outflow tract-time velocity integral.**

| **Quantity** | **Apparent (full cohort)** | **Bootstrap median** | **95 % CI** |
| --- | --- | --- | --- |
| Threshold | -5.0 % | -5.0 % | -28 % to +6 % |
| Sensitivity | 0.58 | 0.60 | 0.20-0.78 |
| Specificity | 0.56 | 0.56 | 0.37-0.92 |

See Methods section for details. CI, confidence interval.

**Supplemental Table 3. Baseline predictors of decrease ≥5% in left ventricular outflow tract-volume time integral (LVOT-VTI) over 12 month.**

| **Variables** | **Univariable analysis** | | | **Multivariable analysis** | | |
| --- | --- | --- | --- | --- | --- | --- |
|  | **p** | **OR** | **95% CI** | **p** | **OR** | **95% CI** |
| Age | 0.049 | 1.017 | 1.000-1.034 | 0.989 | - | - |
| Male sex | 0.210 | - | - | - | - | - |
| *TTR* genotype (wild-type, V122I, non-V122I) | 0.043 | n/a | n/a | 0.108 | - | - |
| Comorbidities | | | | | | |
| Atrial fibrillation | 0.382 | - | - | - | - | - |
| Ischaemic heart disease | 0.418 | - | - | - | - | - |
| Diabetes mellitus | 0.640 | - | - | - | - | - |
| Hypertension | 0.062 | - | - | - | - | - |
| NYHA class | 0.396 | - | - | - | - | - |
| NAC stage | 0.033 | n/a | n/a | 0.003 | n/a | n/a |
| NT-proBNP | 0.362 | - | - | - | - | - |
| eGFR | <0.001 | 0.980 | 0.972-0.989 | - | - | - |
| HF therapies | | | | | | |
| Loop diuretic | 0.017 | 1.525 | 1.079-2.155 | 0.094 | - | - |
| MRA | 0.013 | 1.443 | 1.082-1.926 | 0.031 | 1.423 | 1.033-1.959 |
| ACEi/ARB/ARNI | 0.211 | - | - | - | - | - |
| SGLT2i | 0.139 | - | - | - | - | - |
| Beta-blocker | 0.833 | - | **-** | - | - | - |
| PPM | 0.595 | - | - | - | - | - |
| ICD | 0.196 | - | - | - | - | - |
| CRT | 0.768 | - | - | - | - | - |
| Baseline echo | | | | | | |
| IVS (mm) | 0.880 | - | - | - | - | - |
| PW (mm) | 0.105 | - | - | - | - | - |
| RWT | 0.203 | - | - | - | - | - |
| LV mass (g) | 0.179 | - | - | - | - | - |
| LVEF (%) | 0.291 | - | - | - | - | - |
| LV GLS (%) | 0.199 | - | - | - | - | - |
| LVOT-VTI (cm) | <0.001 | 1.116 | 1.081-1.151 | <0.001 | 1.146 | 1.107-1.186 |
| E/e’ ratio | 0.208 | - | - | - | - | - |
| TAPSE (mm) | 0.056 | - | **-** | - | - | - |
| RV FAC (%) | 0.875 | - | - | - | - | - |
| sPAP (mmHg) | 0.139 | - | - | - | - | - |

Logistic regression analysis. Significant p values are reported in bold. Odds ratio (OR) and 95% confidence interval (CI) values were not calculated for categorical variables with more than two variables, namely *TTR* genotype and National Amyloidosis Centre (NAC) stage. ACEi/ARB/ARNI, angiotensin converting enzyme inhibitor/angiotensin receptor blocker/angiotensin receptor/neprylisin inhibitor; CI, confidence interval; CRT, cardiac resynchronization therapy; eGFR, estimated glomerular filtration rate; FAC, fractional area change; GLS, global longitudinal strain; ICD, implantable cardioverter defibrillator; IVS, interventricular septum thickness; LV, left ventricular; LVEF, left ventricular ejection fraction; MRA, mineralocorticoid receptor antagonist; n/a, not available; NT-proBNP, N-terminal pro–B-type natriuretic peptide; NYHA, New York Heart Association; OR, odds ratio; PPM, permanent pacemaker; PW, posterior wall; RV, right ventricular; RWT, relative wall thickness; sPAP, systolic pulmonary artery pressure; SGLT2i, sodium glucose cotransporter 2 inhibitor; SV, stroke volume; TAPSE, tricuspid annular plane systolic excursion.

**Supplemental Table 4. Changes in echocardiographic parameters in patients developing a decrease in left ventricular outflow tract-volume time integral (LVOT-VTI) ≥5% over 12 month.**

| **Δ%** | **LVOT-VTI decrease ≥5%**  **n=377 (50.1%)** | **No LVOT-VTI decrease ≥5%**  **n=375 (49.9%)** | **p** |
| --- | --- | --- | --- |
| IVS (mm) | +1.65 (-6.68 to +11.22) | +2.53 (-7.66 to +11.09) | 0.948 |
| PW (mm) | +1.80 (-12.62 to +15.29) | +2.81 (-10.33 to +21.18) | 0.405 |
| RWT | +1.19 (-12.72 to +18.61) | +2.93 (-14.37 to +22.81) | 0.650 |
| LV mass (g) | +1.47 (-11.12 to +15.14) | +4.24 (-9.44 to +20.76) | 0.134 |
| LVEF (%) | -8.22 (-22.10 to +7.11) | +6.00 (-9.15 to +19.39) | **<0.001** |
| LV GLS (%) | -5.56 (-26.14 to +9.54) | +0.94 (-13.22 to +27.43) | **0.008** |
| LVOT-VTI (cm) | -18.74 (-28.58 to -11.34) | +11.38 (+3.39 to +24.12) | **<0.001** |
| E/e’ ratio | +3.53 (-10.95 to +23.75) | +6.36 (-6.56 to +23.28) | 0.204 |
| TAPSE (mm) | -6.05 (-30.22 to +19.30) | +2.26 (-19.16 to +35.93) | **<0.001** |
| RV FAC (%) | -13.30 (-39.87 to +28.21) | -0.92 (-31.44 to +32.83) | 0.187 |
| sPAP (mmHg) | +3.15 (-13.89 to +26.80) | +4.23 (-16.14 to +33.18) | 0.874 |

Significant p values are reported in bold. FAC, fractional area change; GLS, global longitudinal strain; IVS, interventricular septum; LV, left ventricular; LVEF, left ventricular ejection fraction; LVOT-VTI, left ventricular outflow tract-velocity time interval; PW, posterior wall; RV, right ventricular; RWT, relative wall thickness; sPAP, systolic pulmonary artery pressure; SV, stroke volume; TAPSE, tricuspid annular plane systolic excursion.
